# Supplementary material for: Sorting at embryonic boundaries requires high heterotypic interfacial tension
Source: Nat Commun. 2017 Jul 31;8:157. doi: 10.1038/s41467-017-00146-x (PMC5537356; doi:10.1038/s41467-017-00146-x)
Supplement: Supplementary file 2 — Supplementary Software 1 [file 41467_2017_146_MOESM2_ESM.zip › PottsModel/SrcPottsModel/doc/simulations/package-tree.html]

simulations Class Hierarchy


JavaScript is disabled on your browser.


Skip navigation links


- Overview
- Package
- Class
- Use
- Tree
- Deprecated
- Index
- Help

- Prev
- Next

- Frames
- No Frames

- All Classes

# Hierarchy For Package simulations

Package Hierarchies:

- All Packages

## Class Hierarchy

- java.lang.Object
  - engine.Simulation
    - simulations.BoundarySimulation
    - simulations.DefaultSimulation
    - simulations.DITHSimulation
    - simulations.EndoSimulation
    - simulations.FourByFourSimulation
    - simulations.GenerateRandomLattices
    - simulations.GranerGlazierSimulation
    - simulations.NegativeControl
    - simulations.SingleCell

Skip navigation links


- Overview
- Package
- Class
- Use
- Tree
- Deprecated
- Index
- Help

- Prev
- Next

- Frames
- No Frames

- All Classes
